# Factors affecting healthcare utilization for low back pain among nurses in Gondar town, northwest Ethiopia, 2018: a cross-sectional study

**DOI:** 10.1186/s13104-019-4231-2

**Published:** 2019-03-29

**Authors:** Tesfaye Hambisa Mekonnen, Dawit Getachew Yenealem

**Affiliations:** Department of Environmental and Occupational Health and Safety, Institute of Public Health, College of Medicine and Health Sciences, University of Gondar, P.O.Box 196, Gondar, Ethiopia

**Keywords:** Low back pain, Nurses, Healthcare utilization, Ethiopia

## Abstract

**Objective:**

The objective of this study was to investigate level and factors affecting healthcare utilization for low back pain (LBP) among nurses in Gondar town, Ethiopia. A healthcare-based cross-sectional study was conducted from March to April 2018. We included 422 nurses using stratified sampling technique. A binary logistic regression analysis was performed using SPSS version 20 to identify factors associated with healthcare utilization.

**Results:**

A total of 422 nurses with 100% response rate participated in this study. The majority, 54.8% (N = 277) were females. Mean age was 21.54 (SD ± 4.99) years. Of the 64% (N = 270) LBP sufferers, 25.4% (N = 107) [95% CI (21.1, 29.6)] had used healthcare services at least once in the previous 12 months. Sex [AOR: 1.82; 95% CI (1.03, 3.43)], educational level [AOR: 1.13; 95% CI (1.01, 3.40], perceived disability [AOR: 2.11; 95% CI (1.66, 3.20)], and perceived severity [AOR: 2.06; 95% CI (1.27, 3.51)] were associated factors. This study reveals that healthcare service utilization for low back pain was not common practices among nurses. Strategies that focus on nurses’ gender and educational level differences should be in place to promote care usage for low back pains.

## Introduction

The health and economic burdens of occupational-related low back pain (LBP) are often superseding. It has been shown that LBP causes an estimated 21.7 million disability adjusted life years (DALYs) accounting for about one-third of all occupational disabilities [1]. The burden of LBP also entails a profound negative impact on the quality of life, productivity, and work performance [2–4]. Globally, there has been a plethora of research conducted on the prevalence and risk factors arousing LBP among healthcare workers, particularly nurses [5–10].

Despite the ample studies available on the prevalence and risk factors of LBP, information is scant on the practices of seeking healthcare services for the ailments among nurse professionals [11]. It has been showed that experiencing back pain for more than 6 weeks is a serious health problem owing to certain kinds of treatment services [12, 13]. Depending on the conditions, numerous treatment options are available for LBP. Self-care, such as taking the necessary rests, ice or heat, massage, pain relievers, and gentle stretches are usually eminent treatment strategies [12]. The use of drugs, like non-steroidal anti-inflammatory drugs, muscle relaxant, analgesics, and steroidal treatment [4] and physical/exercise therapy, such as ultrasound, diathermy, yoga, chiropractic manipulation, and acupuncture have been showed to be beneficial to treat chronic or recurrent low back pain disorders [3, 12, 14–17].

Despite the treatment options available, care seeking for LBP among nurses is often peculiar. For instance, a study done in the Netherlands indicated that the majority of LBP sufferers had not sought treatment [18]. Literature illustrates that variations in the level of treatment utilization for LBP ranges from 4.54 to 28% [19, 20]. Another report demonstrated that only 16% of medical care was sought for low back pain [21]. Moreover, various investigations have exhibited that there are a number of notable factors, including sex, perceived disability [20, 22, 23], duration of back pain [19], and perceived severity [23] which can affect health service utilization for back pain conditions. It has also been shown that the decisions to seek care for LBP is related to economic and occupational risk factors [19] and the nature of pain [7, 24].

To date, in Ethiopia, evidence is meager on the status and a number of factors affecting healthcare utilization due to LBP among nurse professionals. The current study is, therefore, aimed to determine the status and identify factors affecting healthcare utilization due to back pain among nurses in Gondar town, northwest Ethiopia.

## Main text

### Methods

#### Study design, setting and period

A healthcare-based cross-sectional study was conducted from March to April 2018. Healthcare facilities in Gondar town were the study setting. The town is located 748 km to the northwest of Addis Ababa, the capital of Ethiopia. In the town, there are two hospitals (public and private), employing more than 600 nurse professionals, five public health centers, and more than ten private clinics, employing more than 391 nurse healthcare workers. We included the two hospitals purposively and 8 randomly selected health centers (five from private and 3 from public) to attain the required sample.

### Populations and sample size

Nurses who had worked for at least 12 months prior to the study period were included and we excluded those on sick, annual, maternity and other leaves. A single population proportion formula was employed to calculate the sample size with n, (minimum sample size), z = 1.96 (critical value) with 95% CI, p = 50% (the proportion of healthcare utilization for low back pain), and d = 5% (margin of error). Hence, n = ((z^2^) (p) (1_p)) ÷ d^2^; n = (1.96)^2^ (0.5) (1_0.5) ÷ (0.05)^2^ = 384 and assuming a 10% no response, the final sample was = 384 + 38.4 = 422. We considered the 50% proportion of care utilization because there has not been similar previous study accessed on the topic in Ethiopia. Healthcare workers were stratified according to the type of health facility (private & public).

#### Data collection **tools** and techniques

The data collection was conducted using a structured and interviewer-administered questionnaire. We assessed the prevalence of low back pain by the standardized Nordic Questionnaire [25]. Perceived severity and disability of low back pain were evaluated according to the Von Korff et al. [26]. The 10-items generic job satisfaction scale questionnaire was used to assess the satisfaction of nurses with their jobs [27]. We also assessed job stress using the 8-items job stress scale questionnaire [28].

### Data analysis and quality control

Data collection tool was developed in English and translated to Amharic, the local language, and retranslated to English by language experts. Three well-experienced data collectors and two supervisors involved in the data collection. The data collectors and supervisors had taken training and orientation for 2 days. We also conducted a pre-test at the neighboring Kola Diba hospital on 5% of the sample. Based on the pretest, we minimized the amount of questions and corrected some other ambiguities.

Data were entered into EPI info version 7.1.5.2 and exported to SPSS version 20 software for analysis. We described the results using frequencies, percentages, means, and standard deviations. The reliability of data collection instrument was tested and we found a reliable Cronbach’s Alpha score (Cronbach’s Alpha = 0.88). A variable inflation factor (VIF) was employed to check the multicollinearity and found no evidence of collinearity (VIF < 5). A multivariable logistic regression model was used to identify potential confounders. We verified goodness of fit model using the Hosmer and Lemeshow test (*p* value > 0.05). The model was also evaluated using the area under the receiver operating characteristics (ROC) curve. Hence, the test showed that 89.1% of the positive outcome of interest/care utilization/could be correctly predicted by the model (AUC = 0.891; p = 0001).

### Operational definition

Perceived severity: A pain intensity score of ≥ 50 or < 3 disability points [26].

Perceived disability: A pain disability point score of 3–6 points [26].

Stressed worker: The workplace stress scale score of 21 or above [28].

Job satisfied worker: The generic job satisfaction scale score of 32 or above [27].

### Results

#### Socio-demographic characteristics

**Fig. 1:**
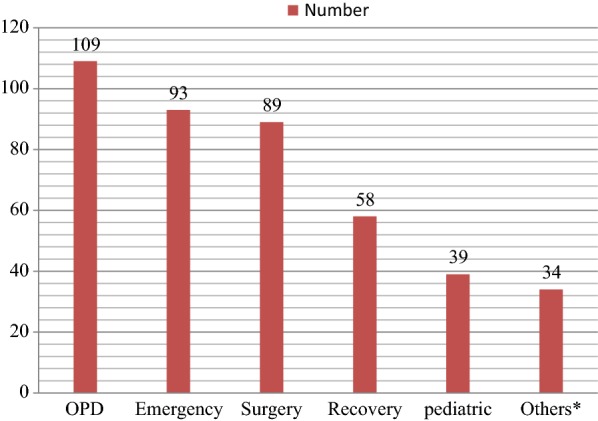
Distribution of nurses by working department in Gondar town health facilities, Ethiopia, 2018 (N = 422)

Response rate was 100%. The majority, 53.8% (N = 227) were females. About 72.7% (N = 307) were in the age group of 25–35 years. The mean age was 21.54 (SD ± 4.499) years. Of the respondents, 77.9% (N = 329) were Amhara and 54.3% (N = 229) were married. The majority of the participants, 55.4% (N = 234) were diploma graduates (Table 1). High proportions, 86.3% (N = 364) of the participants were permanent employees, 51% (N = 219) worked more than 8 h per day, 73.5% (N = 310) said they worked over time, 58.3% (N = 246) worked night shift, 5.7% (N = 24) day shift, 31% (N = 131) served on both shifts, and 5% (N = 21) no shift. Over three-fourth, 76.3% (N = 322) and 38.4% (N = 162) of the participants reported that there was pre-employment and periodic medical examination services at their work, respectively. A limited proportions, 28.8% (N = 109) of the respondents marked that they worked in outpatient departments (OPD) (Fig. 1).

**Table 1:** Socio-demographic characteristics of nurses in Gondar town,, Ethiopia, 2018 (N = 422)

Variables	Frequency	Percent (%)
Sex		
Male	195	46.2
Female	227	54.8
Age		
≤ 24	36	8.5
25–35	307	72.7
> 35	79	18.7
Religion		
Orthodox	250	59.2
Muslim	118	28.0
Protestant	54	12.8
Marital status		
Single	159	37.8
Married	229	54.2
Separated/divorced/widowed	34	8.0
Educational level		
Diploma	234	55.4
First degree (BSc)	124	29.3
Masters degree	64	15.2
Monthly salary in BIRR		
≤ 3500	148	35.1
3501–4500	73	17.3
> 4500	201	47.6

#### Level of health care utilization due to low back pains

Out of the reported 64% (N = 270) low back pain complaints, 25.6% (n = 69) [95% CI (20.1, 30.6)] had sought treatment services at least once in the previous 12 months. Slightly over half, 65.2% (n = 45) of the respondents who sought medical care were females and 46.4% (n = 32) of them were > 35 years of age, while 23.2% (n = 16) of them were 25–35 years old. Twenty-six percent (n = 18) of the care seekers for low back pains were diploma and 43.5% (n = 30) were first degree (Bachelor of Science) graduates. Regarding working hours, 58% (n = 40) of interviewees reported they worked > 8 h per day, whereas 42% (n = 29) of them indicated they had worked ≤ 8 h per day. About, 32% (n = 22) of the respondents exhibited they perceived their LBP complaints as a low disabling, while 68% (n = 47) of them showed they perceived their symptoms as a high disabling pain. Seventy-eight percent of the nurses who sought treatment for LBP (n = 54) demonstrated their pain as a severe, whereas 21.7% (n = 15) of them indicated not. Among the participants having had utilized treatment services for low back pains, 62.3% (n = 43) clarified that they were not satisfied with their current jobs.

#### Factors associated with healthcare utilization due to low back pains

Sex, age, educational status, work experience, overtime work, working hours, duration of low back pain, job stress, job satisfaction, perceived severity, and perceived disability were the factors associated with healthcare utilization for low back pain in a bivariate analysis.

In the multivariable logistic regression analysis, sex, educational level, perceived severity of low back pain, and perceived disability were the factors markedly affected seeking care services for LBP. Females were 1.28 times more likely to seek healthcare services due to their low back pain than their male counterparts [AOR: 1.28; 95% CI (1.03, 3.43)]. Participants who had held masters’ degrees educational levels were 1.13 times more likely to seek treatment than who had diploma [AOR: 1.13; 95% CI (1.01, 3.40)]. The odds of health care utilization due to low back pain increased by a factor of 2.11 among participants who perceived their low back pain as a disabling than those who didn’t perceive it as disabling pain [AOR: 2.11; 95% CI (1.66, 3.30)]. The likely hood of utilizing health care services was 2.06 times higher among respondents who perceived their low back pain as a severe than those who didn’t [AOR: 2.06; 95% CI (1.27, 3.51)] (Table 2).

**Table 2:** Factors affecting HCU for LBP among nurses, Gondar town, Ethiopia, 2018 (N = 270)

Variables	Healthcare utilization for LBP		COR (95% CI)	AOR (95% CI)	*p*-*value*
	Yes	No			
Sex					
Male	24	100	1	1	
Female	45	101	1.85 (1.05, 3.27)	1.28 (1.03, 3.43)	0.001*
Age					
< 24 years	21	51	1	1	
25–35 years	16	101	0.38 (0.12–0.94)	0.24 (0.01–0.59)	0.110^+^
> 35 years	32	49	1.60 (1.05–.2.51)	1.31 (0.03–2.03)	0.071^+^
Educational level					
Diploma	18	87	1	1	
First degree (Bsc)	30	80	1.81 (0.99, 3.40)	1.12 (0.73, 2.17)	0.061^+^
Masters degree	21	34	2.98 (0.76, 3.96)	1.13 (1.01, 3.40)	0.003*
Job stress					
Not stressed	22	97	1	1	
Stressed	47	104	1.99 (1.11, 3.54)	1.08 (1.21, 2.17)	0.078^+^
Work experience					
≤ 5 years	47	99	2.20	1.40 (0.03, 3.21)	0.056^+^
> 5 years	22	102	(1.23, 3.92)	1	
Working hours per day					
≤ 8 h	29	98	1	1	
> 8 h	40	103	1.31 (1.02, 6.55)	1.17 (0.63, 5.12)	0.073^+^
Overtime					
Yes	56	161	1.07 (0.53, 2.14)	1.01 (0.07, 4.30)	0.081^+^
No	13	40	1	1	
Job satisfaction					
Satisfied	26	91	1	1	
Not satisfied	43	110	1.37 (1.78, 2.39)	1.14 (0.35, 3.44)	0.031^+^
Perceived disability					
Low disability	22	107	1	1	
High disability	47	94	2.43 (1.83, 3.51)	2.11 (1.66, 3.20)	0.001*
Perceived severity					
Severe pain	54	120	2.43 (1.28, 4.60)	2.06 (1.27, 3.51)]	0.001*
Not severe pain	15	81	1	1	
Duration of LBP					
0–7 days	10	63	1	1	
8–30 days	31	73	2.68 (0.17, 2.82)	1.71 (0.12, 2.01)	
> 30 but not every day	22	43	3.22 (0.13, 5.72)	2.27 (0.23, 2.81)	
Every day	6	22	1.72 (0.18, 4.78)	1.37 (0.44, 3.09)	0.08*

AOR, adjusted odds ratios; CI, confidence interval; COR, Crude odds ratios; HCU, Healthcare utilization; BSc, Bachelor of Science; LBP, low back pains

1 = represents reference categories; ^**+**^=significant in a bivariate analysis;* = significant in a multivariable analysis

### Discussion

We first assessed the prevalence of low back pains, to investigate status and the factors affecting health service usage, out of the sufferers. The 12 months prevalence of low back pain was 64% (N = 270) [95% CI (59.5, 68.5)]. Our finding demonstrates that, out of the victims, 25.6%; [95% CI (20.1–30.6)] had used healthcare services at least once in the previous 12 months. This finding was relatively equivalent to that of a study conducted in Switzerland (28%) [20]. The possible reason could be due to similarities in the nature of healthcare facility working environments. But our result was higher than that of a study done in Nigeria (11.9%) [29]. A possible explanation for this difference might be due to the variations in data collection methods and target populations. We found a lower prevalence of healthcare utilization for back pain compared to the reports from Malaysia (34.1%) [10], Bangladesh (35%) [30] and (36.2%) [31], Norway (43%) [32], Netherland (44%) [33], India (45%) [34], and the United States (84.0%) [35]. These disparities might be because of differences in accessibility of health services, level of economic capacity to utilize medical services, variations in perceiving severity and disability of back pains, workplace illness and injury management and reporting procedures.

Our study demonstrated that sex was a significant factor for healthcare utilization due to LBP. According to this study, being female was more likely to increase the odds of seeking medical care because of low back pain than being male. This was in line with the findings of previous studies [9, 22, 36]. This could be due to the fact that females are usually cognizant of the benefits of using healthcare services. Another possible explanation might be that nursing is usually dominated by female professionals, which is true in the current study.

In this study, we found a considerable association between healthcare seeking due to low back pain and level of education. Previous studies reported similar findings [32, 37]. Education increases peoples’ level of awareness and knowledge about the prevention and control of specific health problems.

The current study indicated that perceived severity of LBP remarkably affected seeking treatment services. Our result corroborated to other reports [20–22, 32, 33]. A possible suggestion is that severe back pain might negatively affect health status and the health professionals (nurses) become aware of the conditions after it has become serious. A previous study supports this explanation [22]. Perceived disability is the other factor that significantly predicted decision to seek healthcare utilization due to low back pain. This finding corresponds to the other studies [20, 22, 32, 33]. This could be explained by the awareness and knowledge of nurses about the ultimate consequences of their ailments that predisposes them to use healthcare, probably when their condition begins to manifest impairments in their daily activities.

### Conclusions

This study reveals that healthcare service utilization for low back pain is not common practices among nurses. Strategies that focus on nurses’ gender and educational level differences should be in place to promote care usage for LBP. Procedures that can address pains related to back pains is also an imperative approach to encourage nurses’ care seeking for low back pain.

## Limitations

The self-report data collection method employed in the current study might be one of the suspected limitations, leading to underestimation (due to recall bias). It might also be problematic to judge a temporal-relationship between healthcare usage due to LBP and the factors that affect it only by using a cross-sectional design. Moreover, it might be uncertain to generalize the findings of the study, as the study was dealt with only a specific segment of workforces. Therefore, future investigations with a large sample from various sectors and strong study designs, such as longitudinal, are greatly suggested.

## Abbreviations

AOR: adjusted odds ratios
BMI: body mass index
BSc: Bachelor of Science
CI: confidence interval
COR: Crudes odds ratios
HCU: Health care utilization
KM: kilometer
LBP: low back pain
MPH: Masters of public health
OR: odds ratios
SD: standard deviations
SPSS: Statistical Package for Social Sciences
